# RNA-seq Profiling Showed Divergent Carbohydrate-Active Enzymes (CAZymes) Expression Patterns in *Lentinula edodes* at Brown Film Formation Stage Under Blue Light Induction

**DOI:** 10.3389/fmicb.2020.01044

**Published:** 2020-05-27

**Authors:** Xiying Huang, Runji Zhang, Yijie Qiu, Haibing Wu, Quanju Xiang, Xiumei Yu, Ke Zhao, Xiaoping Zhang, Qiang Chen, Petri Penttinen, Yunfu Gu

**Affiliations:** ^1^Department of Microbiology, College of Resources, Sichuan Agricultural University, Chengdu, China; ^2^Department of Livestock and Fisheries, Mianyang Academy of Agricultural University, Mianyang, China

**Keywords:** RNA-seq, CAZymes, *Lentinula edodes*, brown film formation, blue light

## Abstract

*Lentinula edodes* (shiitake mushroom) is one of the most important edible mushrooms worldwide. The *L. edodes* cultivation cycle includes a unique developing stage called brown film formation that directly affects the development of primordium and the quality of fruiting body. Brown film formation is induced by light, especially blue light. To promote our understanding of the role of blue light in brown film formation mechanisms of *L. edodes*, we used RNA-seq and compared the transcriptomes of *L. edodes* grown under blue light and in dark, and validated the expression profiles using qRT-PCR. Blue light stimulated the formation of brown film and increased the content of polysaccharides in *L. edodes.* Blue light also promoted *L. edodes* to absorb more polysaccharides by enhancing the activities of enzymes. Among the 730 differentially expressed genes (DEGs), 433 genes were up-regulated and 297 were down-regulated. Most of the DEGs were in the oxidoreductase activity group. Pentose and glucuronic acid conversion and starch and sucrose metabolism were the most important pathways in the formation of brown film. A total of 79 genes of DEGs were identified as genes encoding carbohydrate-active enzymes (CAZymes). Fifty-one of the CAZymes genes were up-regulated, suggesting that CAZymes play important roles in brown film formation to provide sufficient nutrition for *L. edodes*. The results will facilitate future functional investigations of the genes involved in the developmental control of *L. edodes*.

## Introduction

*Lentinula edodes* (shiitake mushroom) is one of the most important edible mushrooms in China and worldwide (Yoo et al., 2019). The *L. edodes* cultivation cycle stages include hyphal knot growth, light-induced brown film formation by the mycelia, primordium initiation, and fruiting body development, out of which the brown film formation is considered the most important (Tang et al., 2016; Xie et al., 2018). The brown film formation is a unique developing stage in *L. edodes* cultivation cycle; the depth and the thickness of the brown film directly affect the development of primordium and the quality of fruiting body.

The brown film formation is connected with environmental factors, especially light (Kuratani et al., 2010; Xie et al., 2018). Light affects several mushrooms. For example, blue light can induce differentiation and development of fruiting body in *Pleurotus eryngii* and *Hypsizygus marmoreus* (Hao et al., 2010; Xie et al., 2018), and help to promote the formation of *Ganoderma lucidum* fruiting body and increase the content of triterpenoids in the fruiting body (Hao et al., 2010). Blue light photoreceptors that act as transcriptional regulators in the nucleus have been identified in fungi (Idnurm and Heitman, 2005; Terashima et al., 2005; Yu and Fischer, 2019). The white collar proteins that are parts of the blue light photoreceptors are essential in photomorphogenesis and fruiting body development in *Coprinopsis cinerea* (Kuratani et al., 2010; Tang et al., 2013) and play an important regulatory role in secondary metabolism (Estrada and Avalos, 2008).

The brown film formation entails nutrient accumulation, especially the accumulation of carbohydrates. Obtaining the nutrients from the substrates depends on various enzymes, especially the carbohydrate-active enzymes (CAZymes) that play key roles in the degradation of substrates and connected with the hydrolysis of polysaccharides (Davies and Williams, 2016). Carbohydrate-active enzymes can be divided into six categories based on the motifs and domains associated with catalytic activity: glycosyl hydrolases (GHs), carbohydrate esterases (CEs), polysaccharide lyases (PLs), carbohydrate-binding modules (CBMs), glycosyl transferases (GTs), and auxiliary activities (AAs; Blackman et al., 2015). By degrading carbohydrates in wood, CAZymes can provide *Wolfiporia cocos* with small molecule nutrients that can be directly absorbed (Kubo et al., 2006). However, whether CAZymes are involved in blue light induced brown film formation is still uncertain.

During the past decade, the rapidly developing field of transcriptome profiling using RNA-seq has become increasingly relevant to studies on fungi (Chen et al., 2017; Li et al., 2019; Takano et al., 2019). RNA-seq is a rapid, cost-effective, and high-throughput approach to obtain comprehensive transcriptomic data, and it is particularly attractive for the transcriptome analysis of organisms with no reference genome (Wong et al., 2011; Li et al., 2012). To promote our understanding of the role of blue light in brown film formation mechanisms of *L. edodes* and in inducing the CAZymes, we used RNA-seq and compared the transcriptomes of *L. edodes* grown under blue light and in dark, and validated the expression profiles of the CAZymes genes using qRT-PCR.

## Materials and Methods

### *Lentinula edodes* Cultivation

*Lentinula edodes* ACCC50302 was obtained from the Agricultural Culture Collection of China (ACCC). The culture medium contained a 80% *Quercus acutissima* sawdust, 18% wheat bran, 1% sucrose, and 1% calcium carbonate mixture that was suspended with water 1:1.5 (w:vol). The culture medium was packed into mushroom culture packages weighing each package up to 1 kg, sterilized, and cooled to room temperature. Pre-cultured *L. edodes* was inoculated onto the two sides of the culture packages. To obtain a uniform spread of the hypha in the medium, packages were kept at 20–24°C with 65–70% relative humidity in the dark. After the mycelium had colonized the medium completely within 40 days, the culture packages were moved to 12–22°C with 85–90% relative humidity. The culture packages were divided into blue light treatment (BL) and control groups (CK). The BL was illuminated with blue light LED (455 nm, 80 μmol m^–2^ s^–1^) for 45 days to induce brown film formation, and the CK was maintained for 45 days in darkness. The treatments included three replicates of 20 culture packages. At the brown film formation stage, the mycelia were collected, mixed thoroughly, frozen immediately in liquid nitrogen and stored at −80°C for extraction of RNA and enzyme activity assays. Polysaccharides were extracted from the mycelia using hot water extraction method, and their contents were determined as reported previously (Kozarski et al., 2012).

### RNA Extraction, cDNA Library Construction and Sequencing

Total RNA was isolated using the Trizol Reagent (Invitrogen Life Technologies), after which the concentration and quality were determined using a NanoDrop spectrophotometer (Thermo Scientific Fisher). The integrity of the RNA extracts was confirmed by electrophoresis.

Three micrograms of RNA was used as input material for the RNA sample preparations. Sequencing libraries were generated using the TruSeq RNA Sample Preparation Kit (Illumina, San Diego, CA, United States) (Jain et al., 2020). Briefly, mRNA was purified from total RNA using poly-T oligo-attached magnetic beads (Ozsolak and Milos, 2011). Fragmentation was carried out using divalent cations under elevated temperature in an Illumina proprietary fragmentation buffer. First strand cDNA was synthesized using random oligonucleotides and SuperScript II. Second strand cDNA synthesis was done using DNA Polymerase I and RNase H. Remaining overhangs were converted into blunt ends via exonuclease/polymerase activities and the enzymes were removed. After acetylation of the 3′-ends of the DNA fragments, Illumina PE adapter oligonucleotides were ligated to prepare for hybridization (Wang et al., 2009). To select cDNA fragments of the preferred 200 bp in length, the library fragments were purified using the AMPure XP system (Beckman Coulter, Beverly, CA, United States). DNA fragments with ligated adaptor molecules on both ends were selectively enriched using Illumina PCR Primer Cocktail in a 15 cycle PCR reaction. Products were purified (AMPure XP system) and quantified using the Agilent high sensitivity DNA assay on a Bioanalyzer 2100 system (Agilent). The DNA fragments were sequenced on a Hiseq platform (Illumina) at Shanghai Personal Biotechnology Co. Ltd (Supplementary Figures S1, S2). The raw sequence data were submitted to NCBI Sequence Read Archive (SRA)^1^ with accession number SRP605583.

### Expression Analysis

High-quality clean reads were aligned to the assembled transcriptome using Bowtie2^2^ (Langmead and Salzberg, 2012), and compared to the *L. edodes* reference genome (*Lentinula_edodes*.Lened_assembly 01.dna.toplevel.fa) using Tophat2 (Langmead et al., 2009; Gong et al., 2014). The alignment counts were obtained using RSEM package^3^. Differential gene expression analysis was done and the differentially expressed genes (DEGs) were selected using R package DESeq (Robinson et al., 2010; Love et al., 2014). Genes with *P* < 0.05 and absolute fold change ≥1.0 were taken as differentially expressed as described earlier (Li et al., 2017). *P*-values were adjusted for multiple testing as described earlier (Yang and Jeong, 2018).

### Functional and Pathway Analysis of DEGs

For functional analysis, Gene ontology (GO) analysis of the genes with differential expression was performed in the GO database using Goatools^4^. Genome annotation was based on Ensembl and KEGG (Kyoto Encyclopedia of Genes and Genomes). For pathway enrichment analysis, DEGs were mapped into KEGG pathways using the KOBAS program^5^. *P*-value ≤ 0.05 was taken as the threshold (Li et al., 2015).

### Quantitative Real-Time PCR Validation of RNA-Seq Data

According to the biological role of genes in GO and KEGG analysis and the levels and differences in gene expression between samples, CAZymes genes were selected for validation by quantitative real-time PCR (qRT-PCR). Quantitative real-time PCR was performed on a CFX96 Real-Time System (BIO-RAD) with SYBR green as the fluorescent dye according to the manufacturer’s protocol (Shabbir et al., 2015). The primers used are listed in Supplementary Table S1. Three biological replicates with three technical replicates were performed with GAPDH as the internal control gene as previously described (Zhao et al., 2018).

### Enzyme Assay

To extract extracellular enzymes from the growth media, 10 g wet weight growth medium was suspended in 200 mL of 50 mM sodium acetate buffer (pH 4.8) and centrifuged at 180 rpm for 1 h in ice-bath. Endo- and exo-1,4-beta-glucanase, β-glucosidase, pectin lyase, laccase, and manganese peroxidase (MnP) activities were determined as previously described (Yeo et al., 2007; Songulashvili et al., 2008; MacDonald et al., 2016).

## Results

### Blue Light Causes a Light Stimulated Response Phenotype

**FIGURE 1:**
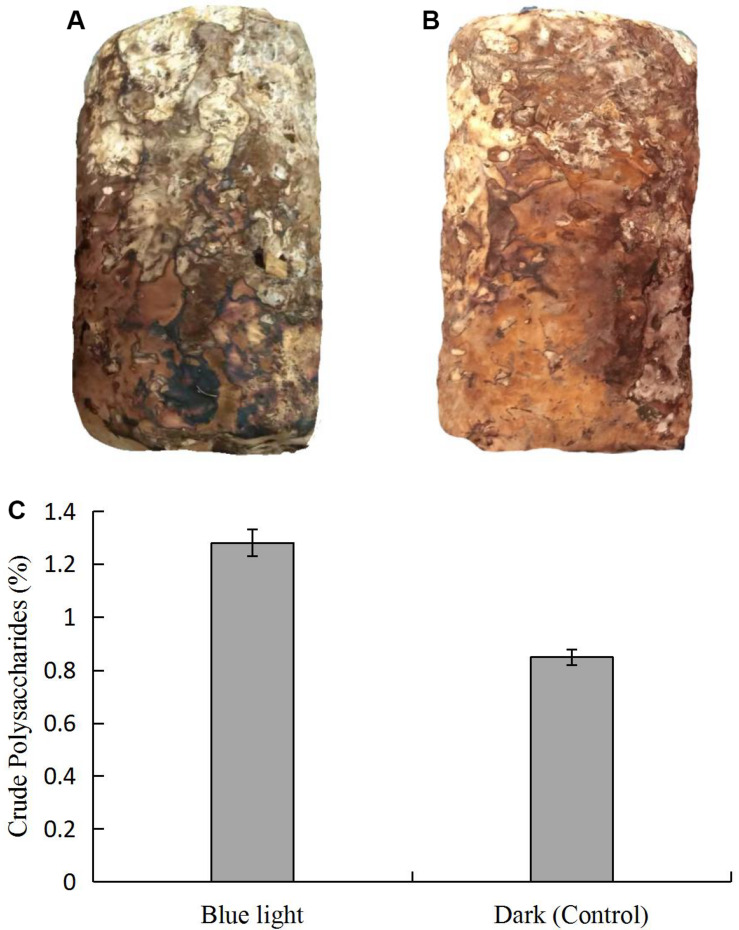
Comparison of *L. edodes* grown **(A)** in dark and **(B)** under blue light, and **(C)** contents of crude polysaccharides in brown film grown in dark and under blue light.

We observed morphological changes in *L. edodes* under standard cultivation conditions after mycelial maturation. With blue light stimulation, color of the *L. edodes* mycelium changed from white to brown in 45 days, which revealed that blue light induced the formation of brown film; the mycelium in the dark grown control also forms a brown film, but the area of brown film was not as big as under blue light (Figures 1A,B). The content of the crude polysaccharides was higher in the brown film with the blue light treatment than in the control (Figure 1C).

### Sequencing and Gene Annotation

After filtering with stringent criteria, we obtained 41.43, 47.04, and 49.15 million reads for the blue light treated *L.edodes*, and 53.06, 48.43, and 44.69 million reads for the control (Table 1 and Supplementary Tables S2, S3). Approximately 65% of the reads were mapped onto the *L. edodes* genome, most of which were uniquely mapped (Supplementary Table S4 and Supplementary Figure S2). Among the genome-mapped reads, about 85% were mapped to gene region, and more than 97% of those were mapped onto exons (Supplementary Table S5). In total, 9,433 genes were recovered.

**TABLE 1:** Numbers of reads and bases in the transcriptomes of *L. edodes* grown under blue light and dark grown control treatment.

Sample	Dark	Blue light
Base number	48,859,319	46,015,817
Clean reads	48,723,001	45,873,228
Mapped reads	34,606,194	29,938,309
Mapped ratio	71.01%	65.26%

### Gene Expression of *Lentinula edodes* Under Blue Light Treatment

Among the 9,433 genes, 730 genes (7.8%) were differentially expressed under the blue light treatment (Supplementary Table S6); 433 genes were up-regulated and 297 down-regulated when compared to the control.

**FIGURE 2:**
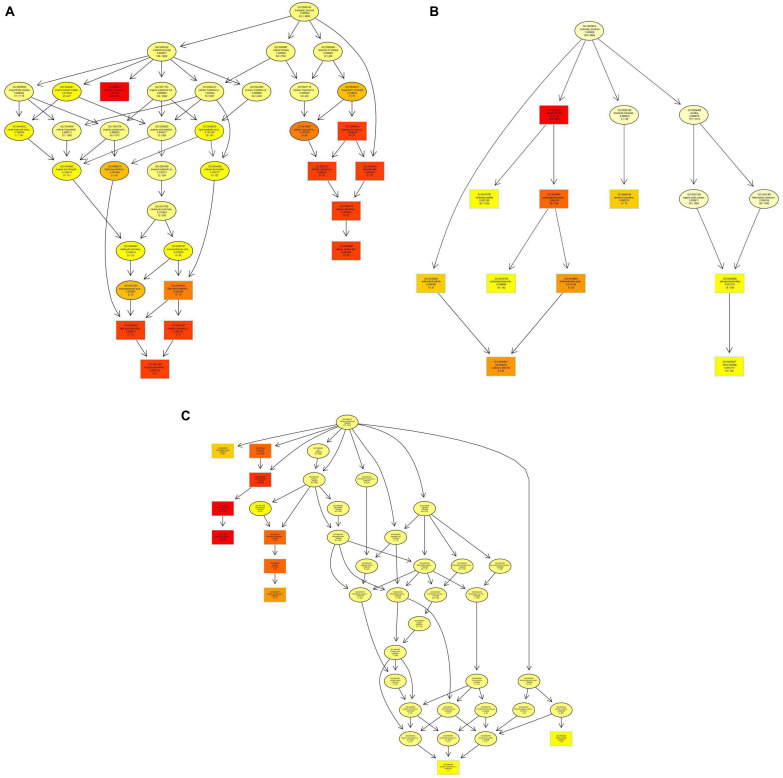
Gene ontology (GO) enrichment in *L. edodes* grown under blue light as compared to the dark grown control treatment. **(A)** Biological processes GO term distribution. **(B)** Molecular functions GO term distribution. **(C)** Cellular components GO term distribution. The GO items are represented by a node, and edges represent an inclusion relationship. The darker the node, the higher the enrichment.

**FIGURE 3:**
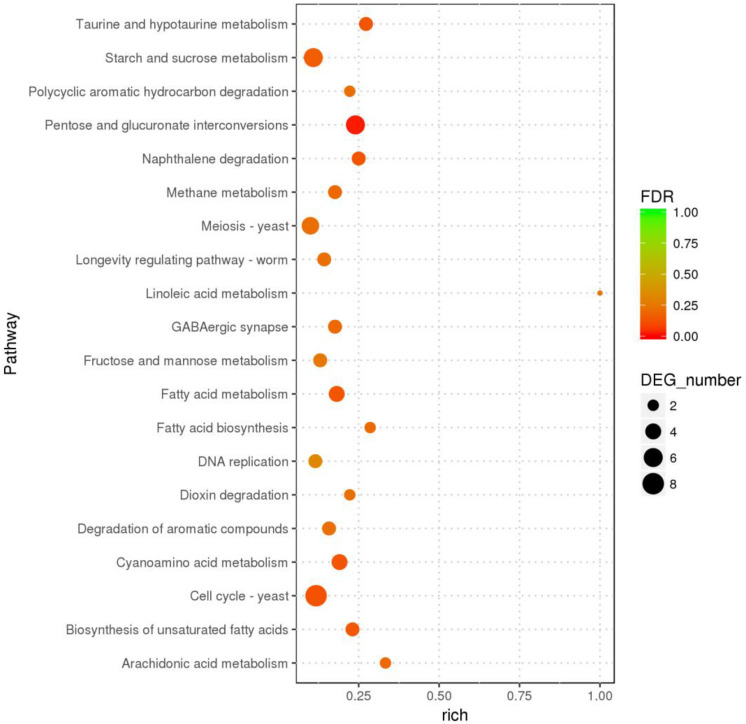
The KEGG pathways with highest number of differentially expressed genes in *L. edodes* grown under blue light as compared to the dark grown control treatment.

In the GO enrichment analysis, the DEGs were classified into 52 cellular components, 161 molecular functions, and 290 biological processes (Figure 2 and Supplementary Table S7), including genes related to cell wall, peroxidase activity, oxidoreductase activity, acting on peroxide as acceptor, and oxylipin biosynthetic process. Among the molecular functions, the most changed DEGs were detected in the oxidoreductase activity group. In the KEGG enrichment analysis, the genes were divided into metabolism, cellular processes, organismal systems, genetic information processing, and environmental information processing categories (Supplementary Table S8). The blue light had greatest effect on metabolic category, especially on pentose and glucuronic acid conversion, cyanoamino acid metabolism, taurine and hypotaurine metabolism, fatty acid metabolism, naphthalene degradation, starch and sucrose metabolism (*P* < 0.05) (Figure 3 and Supplementary Table S8). In addition, the cell cycle and meiosis in cellular processes, GABAergic synapse and longevity regulating pathway in organismal systems were influenced by blue light (*P* < 0.05) (Supplementary Table S8). Metabolism was influenced more than genetic functioning based on the significantly smaller *P*-values (Figure 3 and Supplementary Table S8).

**FIGURE 4:**
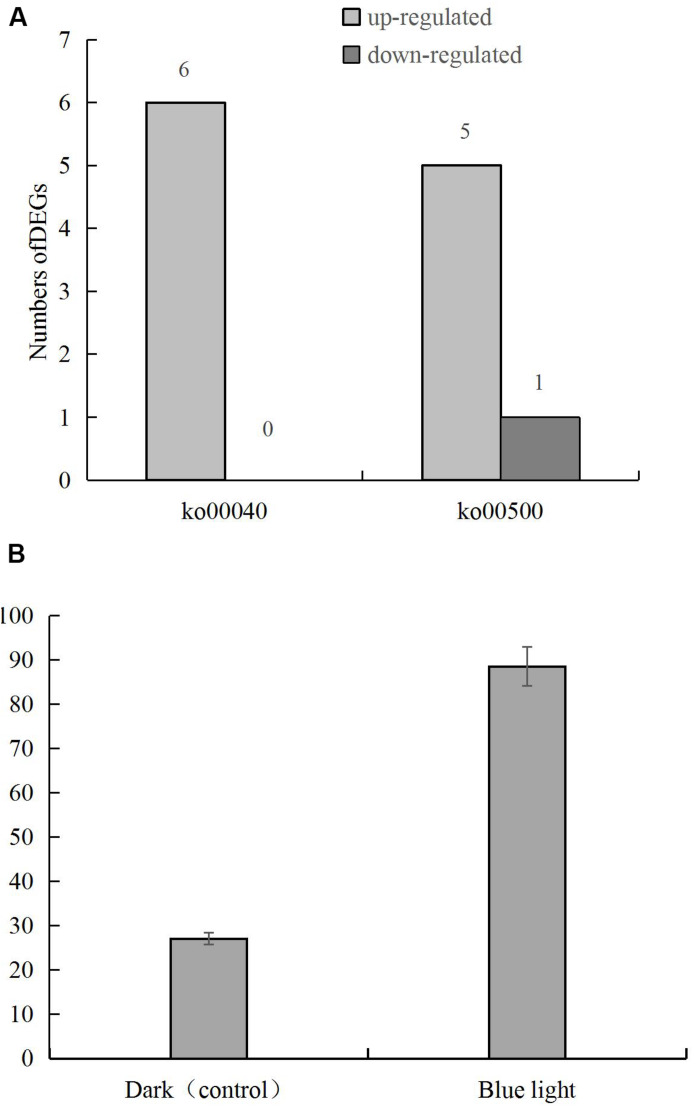
**(A)** The numbers of differentially expressed genes in pentose and glucuronic acid conversion (ko00040) and starch and sucrose metabolism (ko00500) pathways in *L. edodes* grown under blue light as compared to the dark grown control treatment. **(B)** The expression of LENED_005053 gene.

DEGs in the pentose and glucuronic acid conversion and starch and sucrose metabolism pathways showed the highest degree of enrichment and significance. Six genes in the pentose and glucuronic acid conversion pathway (ko00040) were up-regulated, and in the starch and sucrose metabolism pathway (ko00500) 5 and 1 were up-regulated and down-regulated, respectively (Figure 4A), including the up-regulated gene LENED_005053 at the junction of the 2 pathways (Figure 4B). In the specific metabolic pathway, LENED_005053 encodes a glucuronidase [EC: 3.2.1.67] that is involved in the degradation of pectin.

### Blue Light Leads to a Differential Expression of CAZymes Encoding Genes

**FIGURE 5:**
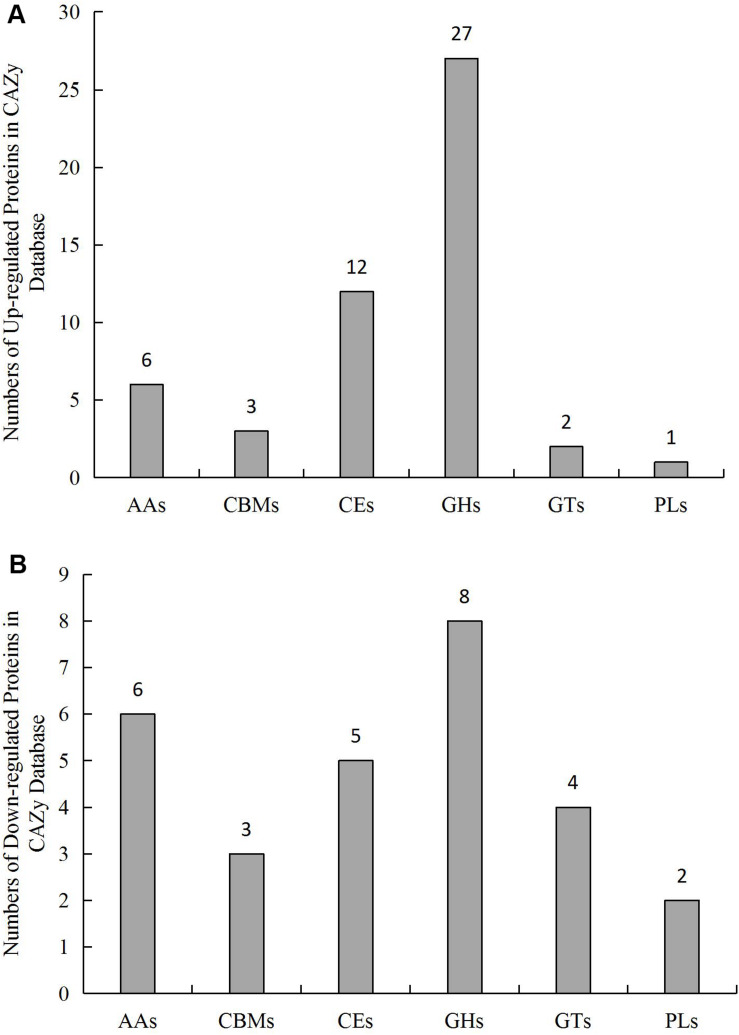
Number and distribution of **(A)** up-regulated and **(B)** downregulated CAZymes genes in *L. edodes* grown under blue light as compared to the dark grown control treatment. AAs, auxiliary activities; CBMs, carbohydrate-binding modules; CEs, carbohydrate esterases; GHs, glycosyl hydrolases; GTs, glycosyl transferases; PLs, polysaccharide lyases.

A total of 712 genes in *L.edodes* transcriptome were identified as CAZymes (Supplementary Table S9). Among these, 253 GHs, 134 AAs, 133 GTs, 121 CEs, 51 CBMs, and 19 PLs were identified (Figure 5 and Supplementary Table S9).

### qRT-PCR Validation of the Differentially Expressed CAZymes

**FIGURE 6:**
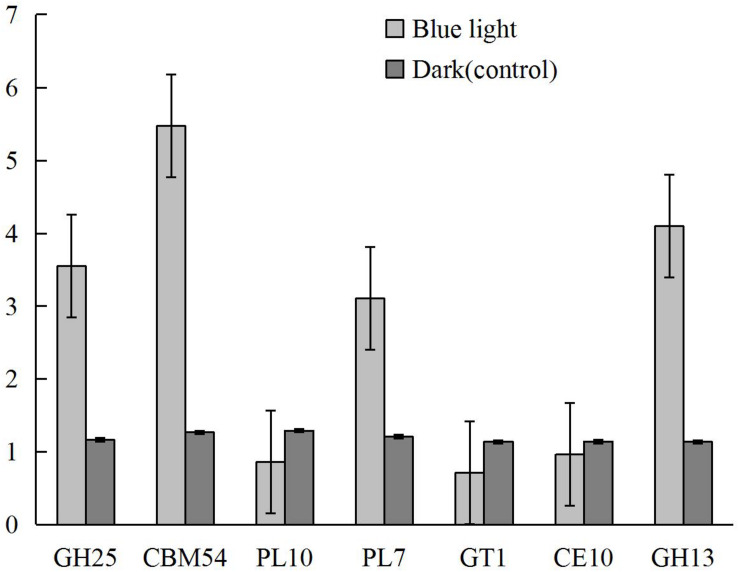
The expression levels of CAZymes CBM54 (LENED_005589), CE10 (LENED_007609), GH13 (LENED_004582), GH25 (LENED_001073), GT1 (LENED_007286), PL10 (LENED_012509) and PL7 (LENED_004566) in *L. edodes* grown under blue light as compared to the dark grown control treatment as determined by qRT-PCR.

Based on the biological roles and the levels and differences in gene expression between samples, seven differentially expressed CAZymes, including CBM54 (LENED_005589), CE10 (LENED_007609), GH13 (LENED_004582), GH25 (LENED_001073), GT1 (LENED_007286), PL10 (LENED_012509), and PL7 (LENED_004566), were selected for qRT-PCR validation. The qRT-PCR gene expression profiles of these genes were in line with the differential expression in RNA-Seq analysis (Figure 6).

### Enzyme Assay

**FIGURE 7:**
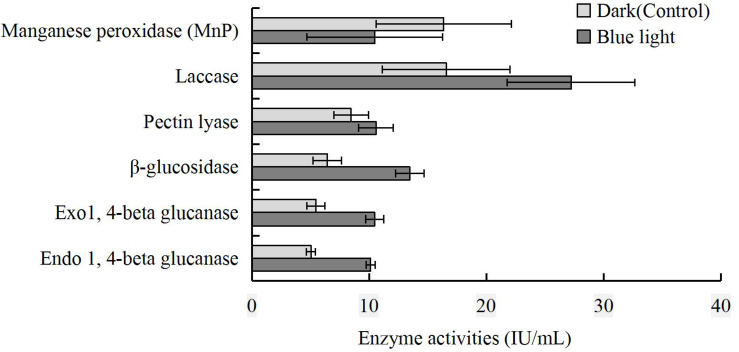
Comparison of MnP, laccase, pectin lyase, β-glucosidase, exo-1,4-beta-glucanase and endo-1,4-beta-glucanase activities at brown film formation stage in *L. edodes* grown under blue light and dark grown control treatment.

To further reveal the impact of blue light on CAZymes, endo- and exo-1,4-beta-glucanase, β-glucosidase, pectin lyase, laccase, and MnP activities in the blue light treatment were compared to those in the control. The activities of endo- and exo-1,4-beta-glucanase, β-glucosidase, pectin lyase, and laccase were higher in the blue light treatment than in the control, and that of MnP was lower (Figure 7).

## Discussion

Light-induced brown film formation by *L. edodes* mycelium is a morphogenesis step peculiar to shiitake mushroom and associated with the quality of the fruiting body. In the process of brown film formation, light is a key environmental factor, and the light quality is more important than quantity (Xie et al., 2018). Blue light was a key signaling component in regulating the gene expression and cellular metabolism of *Drosophila* (Hall et al., 2018), and blue light affects the growth and development of several fungi (Idnurm and Heitman, 2005; Yu and Fischer, 2019). Since knowledge on the molecular mechanisms of brown film formation by *L. edodes* mycelium under blue light is still scarce, we studied the transcriptomes of blue light induced and dark grown control *L. edodes* at the brown film formation stage.

The formation of brown film is the key period for the synthesis and accumulation of polysaccharides in *L. edodes*. In our study, the polysaccharide content of the blue light treated *L. edodes* was higher, indicating that blue light treatment had increased the synthesis of polysaccharides. The synthesis and decomposition of polysaccharides in mushrooms are closely related to pentose and glucuronic acid conversion, and to starch and sucrose metabolism. CAZymes, especially cellulases and hemicellulases, are involved in the hydrolysis of cell wall polysaccharides, and play an important role in substrate degradation processes (Andre et al., 2014; Davies and Williams, 2016). We identified 730 DEGs (433 up-regulated genes and 297 down-regulated genes) in the blue light treated *L. edodes* compared to the dark grown control, out of which 79 were related to CAZymes.

Carbohydrate-active enzymes, including GHs, CEs, PLs, CBMs, GTs, and AAs are involved in carbohydrate metabolism (Cantarel et al., 2009). GHs hydrolyse the glycosidic bond between carbohydrates or between a carbohydrate and a non-carbohydrate moiety (Sathya and Khan, 2015). Among the differentially expressed CAZymes genes, 27 and 8 genes encoding GHs were up-regulated and down-regulated, respectively. The upregulation of GH5, GH17, GH105, and GH109 encoding genes suggested that these enzymes play important roles in providing sufficient nutrition for the growth of *L. edodes*. Similar to Pilgaard (Pilgaard et al., 2019), GH5 genes were likely linked to the cellulase activity and upregulated to help degrade the cellulose and obtain nitrogen. Interestingly, GH5 included also down-regulated genes, demonstrating within enzyme sub-family variation to blue light stimulation.

In GHs that degrade cellulose, hemicellulose, chitin, or arabinolactans, catalytic modules are usually connected to one or more non-catalytic CBMs that function independently (Varnai et al., 2014). In fungi, approximately half of the GH5 proteins harbor a CBM1 module at the N- or C-terminus (Aspeborg et al., 2012). Whether the presence and location of CBM1 module affect the expression of GH5 genes requires further study. In this study, one CBM30 gene, one CBM50 gene and one CBM54 gene were up-regulated, while one CBM1 gene and two CBM13 genes were down-regulated. CBM13 have evolved with a variety of sugar binding specificity and are found in many carbohydrate active enzymes (Fujimoto, 2013). In studies on fungi, the majority of cellulose-binding domains attached to cellulolytic enzymes belong to CBM1 (Chen et al., 2014). On the cellulose surface, the hydrolysis of cellulose are enhanced by CBM1 through increasing the effective enzyme concentration (Takeda et al., 2015). A decrease in the expression of CBM1 may indicate that at the brown film formation stage excessive cellulose degradation enzymes are not required, and that cellulose is not the main degraded substance.

Among the CEs encoding genes, one CE1 gene, one CE4 gene, two CE8 genes, one CE9 gene, four CE10 genes and three CE16 genes were up-regulated, and one CE1 gene, one CE9 gene and three CE10 genes were down-regulated. CE4 is the largest of the CE families, and the structure of CE4 enzymes from a number of bacterial species have been solved (Oberbarnscheidt et al., 2007). CE16 act on hardwood acetyl glucuronoxylan and its fragments generated by endo-β-1,4-xylanases (Biely, 2012). The functions of CE8 and CE9 are still uncovered. Members of families CE1 and CE10 share the common activities of carboxylesterase and endo-1,4-β-xylanase (Zhao et al., 2014), and display a great diversity in substrate specificity. For example, CE10 enzymes may act on non-carbohydrate substrates. The α-helices in CE10 allows binding to specific substrates, such as glutamate or aspartic acid, catalyzing the hydrolytic splitting reaction. Changes in the structure of the enzyme allow different functions after the formation of the active site (Grams and Ospina-Giraldo, 2019), which may explain the inclusion of both up- and down-regulated genes in the CE10 family.

The biosynthesis of disaccharides, oligosaccharides, and polysaccharides involves the action of hundreds of different glycosyl transferases (GTs). Glycosyl transferases can be classified as either retaining or inverting enzymes according to the stereochemistry of the substrates and reaction products (Coutinho et al., 2003; Breton, 2008). Currently many GTs have no known function and few biochemical characteristics. In our research, GT15 and GT49 were up-regulated by blue light, while GT1, GT2, GT8, and GT21 were down-regulated. Among the PL family, which cleave uronic acid-containing polysaccharide chains via α, β-elimination mechanism to generate an unsaturated hexenuronic acid residue and a new reducing end, PL7 gene was up-regulated and PL14 and PL10 genes were down-regulated. PL7 can degrade poly-β-mannuronate and displays endo-β-1, 4-glucuronan lyase activity (Yamasaki et al., 2004). *Alteromonas* sp. 76-1 encoded several alginate cleavage enzymes in the PL7 family with two polysaccharide utilization points (Koch et al., 2019). In this study, blue light may have induced the large-scale synthesis of enzymes with two or more polysaccharide utilization points in the PL7 family, which promotes the degradation of substrates and the synthesis of polysaccharides. Similar to GTs, the specific functions of PLs are yet to be clarified.

The enzymes with AAs accommodate a range of enzyme mechanisms and substrates, including nine families of lignin degradation enzymes and six families of lytic polysaccharide mono-oxygenases (LPMO; Levasseur et al., 2013). In our study, one AA1 gene encoding a laccase, one AA2 gene and four AA3 gene were up-regulated, and one AA6 gene, one AA7 gene and one AA11 gene were down-regulated. When *Staphylococcus aureus* was grown with lignin as the main carbon source, the genes encoding AA3_2, AA3_3 and AA3_4 enzymes were up-regulated (Manavalan et al., 2011). AA3_4, a pyranose oxidase (POx) is found in a number of basidiomycetes (Manavalan et al., 2011). Interestingly, POx is the most abundantly over-expressed protein under carbon-limited conditions (Sutzl et al., 2018). In this research, the four up-regulated AA3 genes belong to AA3_4, the same result as the previous study.

The activity of manganese peroxidase was lower in the blue light treated *L. edod*es than in the control, suggesting that this enzyme was limited at the brown film formation stage. Similar to *P. eryngii* at fruiting body stage (Xie et al., 2018), the activities of endo-1, 4-beta glucanase, exo-1, 4-beta glucanase, β-glucosidase, pectin lyase, and laccase were higher under blue light. Furthermore, the laccase activity was consistent with the up-regulation of AA1 gene.

In summary, the results demonstrated that blue light stimulates the formation of brown film and increases the content of polysaccharides in *L. edodes.* Blue light also promotes *L. edodes* to absorb more polysaccharides by enhancing the activities of enzymes. Among the 730 DEGs, 433 genes were up-regulated and 297 were down-regulated. Most of the DEGs were in the oxidoreductase activity group. Pentose and glucuronic acid conversion and starch and sucrose metabolism were the most important pathways in the formation of brown film. A total of 712 genes were identified as genes encoding CAZymes. 51 of the CAZymes genes were up-regulated, suggesting that CAZymes play important roles in brown film formation to provide sufficient nutrition for *L. edodes*. The functions of these genes need further clarification.

## Data Availability Statement

The datasets presented in this study can be found in the NCBI Sequence Read Archive (SRA, http://www.ncbi.nlm.nih.gov/Traces/sra) under accession number PRJNA605583.

## Author Contributions

YG and HW designed the experiments. XH, RZ, YQ, and HW performed the experiments. XH, YG, and PP wrote and revised the manuscript. QX, XY, KZ, XZ, and QC approved the final version of the manuscript.

## Conflict of Interest

The authors declare that the research was conducted in the absence of any commercial or financial relationships that could be construed as a potential conflict of interest.

## Footnotes

1 http://www.ncbi.nlm.nih.gov/Traces/sra

2 http://bowtie-bio.sourceforge.net/bowtie2/manual.shtml

3 http://deweylab.github.io/RSEM/

4 https://github.com/tanghaibao/GOatools

5 http://kobas.cbi.pku.edu.cn/kobas3/?t=1
