# Supplementary material for: RNA-seq Profiling Showed Divergent Carbohydrate-Active Enzymes (CAZymes) Expression Patterns in Lentinula edodes at Brown Film Formation Stage Under Blue Light Induction
Source: Front Microbiol. 2020 May 27;11:1044. doi: 10.3389/fmicb.2020.01044 (PMC7267012; doi:10.3389/fmicb.2020.01044)
Supplement: Supplementary file 1 [file Data_Sheet_1.docx]

**Supplementary materials**

**RNA-seq profiling shows carbohydrate-active enzymes (CAZymes) expression in *Lentinula edodes* at brown film formation stage under blue-light treatment**

Xiying Huang^1+^, Runji Zhang^1+^, Quanju Xiang^1+^, Xiumei Yu^1^, Ke Zhao^1^, Xiaoping Zhang^1^, Qiang Chen^1^, Petri Penttinen^1^*, Yunfu Gu^1^*

^1^Department of Microbiology, College of Resource Sciences and Technology, Sichuan Agricultural University, Chengdu 611130, China

***Corresponding author**

E-mail: guyf@sicau.edu.cn

Supplementary figure S1:


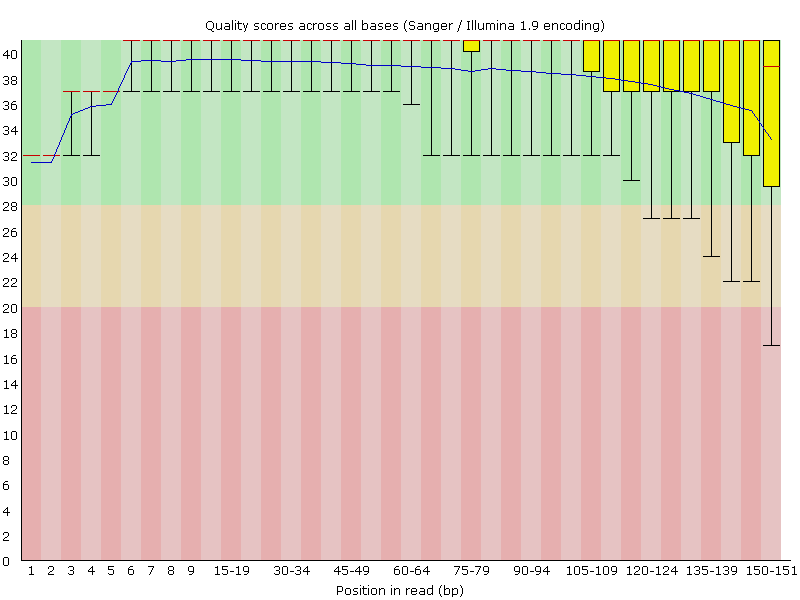


Supplementary figure S2:


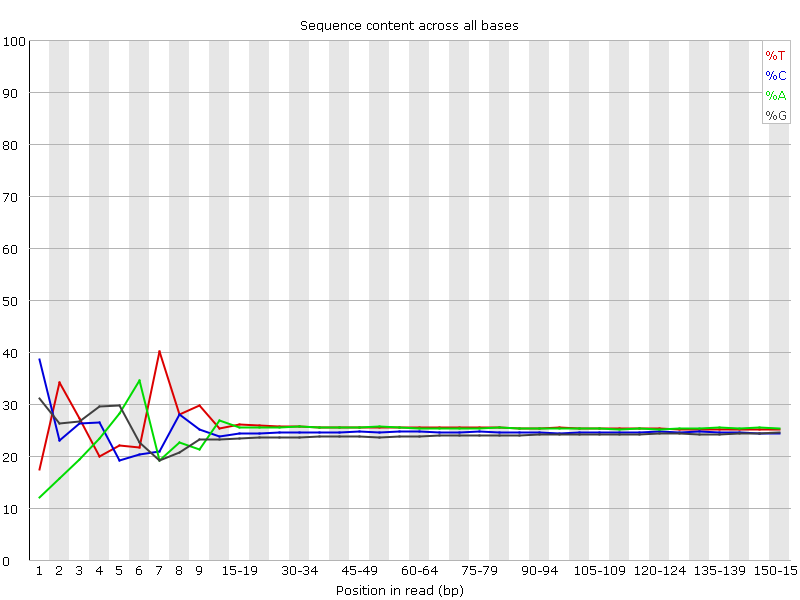


Table S1. the sequences of seven CAZymes genes primer.

| **Target genes** | **Primer sequences** |
| --- | --- |
| LENED_007609 | Forward: 5'ATTCCATCTCCCTTCCGT3' |
|  | Reverse: 5'ATCGTATTTCGTGTTTCGTG3' |
| LENED_007286 | Forward: 5'TATTCCTGCCTACCCTTCC3' |
|  | Reverse: 5'GTTGATTGTGCTCCGATGT3' |
| LENED_012509 | Forward: 5'GACCTTGCGTTCATTTCC3' |
|  | Reverse: 5'CCAGCATTTGACCTTTCTCT3' |
| LENED_001073 | Forward: 5'GAGGGGACCAGTCAGAAAC3' |
|  | Reverse: 5'ATTAGACCAGCATCAGTAGCAC3' |
| LENED_004566 | Forward: 5'TTTCGCCTTCCTACTCCTC3' |
|  | Reverse: 5'CGCACCATCTCCACTTTC3' |
| LENED_004582 | Forward: 5'CAAACGATAGGACAAAGGAAA3' |
|  | Reverse: 5'CCGTGAATGCGATAGATAGA3' |
| LENED_005589 | Forward: 5'ATTCAGAGCCAGTCTTCCTG3' |
|  | Reverse: 5'CGACCATCACCGTCTTCT3' |

Table S2. Summary of the sequencing and assembly.

| **Sample** | **Library Type** | **Reads Length(bp)** | **Raw Reads** | **Raw Data(bp)** | **Q20(%)** |
| --- | --- | --- | --- | --- | --- |
| CK1 | Paired-End | 150 | 53,201,066 | 8,033,360,966 | 97.67 |
| CK2 | Paired-End | 150 | 48,564,420 | 7,333,227,420 | 97.65 |
| CK3 | Paired-End | 150 | 44,812,472 | 6,766,683,272 | 97.64 |
| B1 | Paired-End | 150 | 41,557,912 | 6,275,244,712 | 97.61 |
| B2 | Paired-End | 150 | 47,186,660 | 7,125,185,660 | 97.54 |
| B3 | Paired-End | 150 | 49,302,878 | 7,444,734,578 | 97.59 |

Table S3. Summary of the sequencing data filtered by Q20.

| **Sample** | **Library Type** | **Reads** | **Data(bp)** | **Reads(%)** | **Data(%)** |
| --- | --- | --- | --- | --- | --- |
| CK1 | Paired-End | 53,056,668 | 7,991,306,522 | 99.72 | 99.47 |
| CK2 | Paired-End | 48,426,398 | 7,294,959,810 | 99.71 | 99.47 |
| CK3 | Paired-End | 44,685,936 | 6,730,995,970 | 99.71 | 99.47 |
| BL1 | Paired-End | 41,428,522 | 6,237,708,650 | 99.68 | 99.4 |
| BL2 | Paired-End | 47,037,022 | 7,081,619,394 | 99.68 | 99.4 |
| BL3 | Paired-End | 49,154,141 | 7,400,735,996 | 99.69 | 99.38 |

Table S4. Mapped results of the RNA sequencing data.

| **Sample** | **Useful Reads** | **Map Events Count** | **Total Mapped** | | **Multiple Mapped** | | **Uniquely Mapped** | |
| --- | --- | --- | --- | --- | --- | --- | --- | --- |
|  |  |  | **Reads** | **%** | **Reads** | **%** | **Reads** | **%** |
| CK1 | 53,056,668 | 37,659,996 | 37,867,892 | 71.37 | 207,896 | 0.55 | 37,659,996 | 99.45 |
| CK2 | 48,426,398 | 34,099,237 | 34,279,514 | 70.79 | 180,277 | 0.53 | 34,099,237 | 99.47 |
| CK3 | 44,685,936 | 31,507,887 | 31,671,176 | 70.88 | 163,289 | 0.52 | 31,507,887 | 99.48 |
| BL1 | 41,428,522 | 26,877,986 | 27,022,149 | 65.23 | 144,163 | 0.53 | 26,877,986 | 99.47 |
| BL2 | 47,037,022 | 30,475,648 | 30,634,788 | 65.13 | 159,140 | 0.52 | 30,475,648 | 99.48 |
| BL3 | 49,154,141 | 31,978,447 | 32,157,990 | 65.42 | 179,543 | 0.56 | 31,978,447 | 99.44 |

Table S5. Detailed analysis of gene map counts.

| **Sample** | **Map Events Count** | **Mapped to Gene** | | **Mapped to InterGene** | | **Mapped to Exon** | |
| --- | --- | --- | --- | --- | --- | --- | --- |
|  |  | **count** | **%** | **count** | **%** | **count** | **%** |
| CK1 | 37,659,996 | 31,955,733 | 84.85 | 5,704,263 | 15.15 | 31,075,851 | 97.25 |
| CK2 | 34,099,237 | 29,585,231 | 86.76 | 4,514,006 | 13.24 | 28,757,638 | 97.2 |
| CK3 | 31,507,887 | 27,222,592 | 86.4 | 4,285,295 | 13.6 | 26,464,022 | 97.21 |
| BL1 | 26,877,986 | 22,949,385 | 85.38 | 3,928,601 | 14.62 | 22,296,551 | 97.16 |
| BL2 | 30,475,648 | 26,085,535 | 85.59 | 4,390,113 | 14.41 | 25,349,141 | 97.18 |
| BL3 | 31,978,447 | 27,263,741 | 85.26 | 4,714,706 | 14.74 | 26,485,283 | 97.14 |
